# Characterization of West Nile virus Koutango lineage from phlebotomine sandflies in Kenya

**DOI:** 10.1371/journal.pone.0301956

**Published:** 2024-08-22

**Authors:** Jane Wambui Thiiru, Solomon Langat, Francis Mulwa, Stephanie Cinkovich, Hellen Koka, Santos Yalwala, Samoel Khamadi, Justus Onguso, Nicholas Odemba, Francis Ngere, Jaree Johnson, Timothy Egbo, Eric Garges, Elly Ojwang, Fredrick Eyase

**Affiliations:** 1 Department of Emerging Infectious Diseases, United States Army Medical Research Directorate-Africa, Nairobi, Kenya; 2 Centre for Virus Research, Kenya Medical Research Institute, Nairobi, Kenya; 3 Institute for Biotechnology Research, Jomo Kenyatta University of Agriculture and Technology, Nairobi, Kenya; 4 Global Emerging Infections Surveillance Branch, United States Armed Forces Health Surveillance Division, Silver Spring, Maryland, United States of America; 5 United States Armed Forces Pest Management Board, Silver Spring, Maryland, United States of America; The University of Queensland, AUSTRALIA

## Abstract

The West Nile virus (WNV), primarily transmitted by mosquitoes, is one of the most widespread flaviviruses globally, with past outbreaks occurring in the USA and Europe. Recent studies in parts of Africa, including Kenya, have identified the West Nile virus Koutango lineage (WN-KOUTV) among phlebotomine sandfly populations, however, our understanding of this virus remains limited. This study aimed to characterize WN-KOUTV from phlebotomine sandflies. Sandflies were sampled between 12^th^ -16^th^ March 2021 and 16^th^ -20^th^ March 2023 from six villages each in Baringo and Isiolo Counties, using CDC light traps. Female sandflies were taxonomically identified and pooled based on genus and site of collection. Virus isolation was performed in Vero cells. Viral genomes were determined using next-generation sequencing. Phylogenetic and molecular clock analyses were done to decipher the virus’s evolutionary relationships. Comparative analyses of amino acid sequences were performed to determine variations. Protein modeling in Pymol was conducted to elucidate variations in key protein regions. Evolutionary pressure analysis investigated the selection pressures on the virus. *In vitro* experiments were done to investigate the virus growth kinetics in mammalian Vero E6 and mosquito C6/36 cells. We report the isolation of WN-KOUTV from Salabani in Baringo and Aremet in Isiolo, Kenya. The isolated WN-KOUTVs clustered with previously identified WN-KOUTV strains. Comparative analysis revealed a unique amino acid at NS5 653. The WN-KOUTV lineage as a whole is under purifying selective pressure, with diversifying pressure acting at site NS3 267. The current WN-KOUTV replicated in Vero E6 and C6/36 cells comparable to West Nile virus Lineage 1a, isolated from mosquitoes. Subsequent isolations of WN-KOUTV in phlebotomine sandflies suggest potential vectors, however, vector competence studies would confirm this. Replication in mammalian and insect cell lines suggests there may exist a vector/host relationship. We speculate the close genetic relationship of WN-KOUTV strains from East and West Africa may potentially be enabled by bird migratory routes between the two regions. If proven, this could point to a potential future pandemic pathway for this virus.

## Introduction

West Nile virus (WNV) is an arthropod-borne virus of the genus *Flavivirus*, family *Flaviviridae*, and a member Japanese Encephalitis serocomplex [1]. It was originally isolated in 1937 from the blood of an adult female in Uganda, during routine surveillance for yellow fever virus [2]. Since its initial emergence, WNV has become the most extensively distributed flavivirus, spanning a vast geographic range that encompasses Africa, the Middle East, Europe, Asia, and the Americas [3]. WNV is maintained in an enzootic transmission cycle involving virus reservoir wild birds, mosquito vectors, as well as final or incidental hosts including humans and horses, which are both dead-end hosts [4]. Although several other mosquito species have been suggested to be vectors of WNV, Culex mosquitoes are the primary competent vectors [5]. Birds of orders Passeriformes and Charadriiformes are highly competent hosts that play a crucial role as carriers, amplifiers, and reservoirs of the virus [6]. Approximately, 80% of human cases infected with WNV typically exhibit no symptoms, while around 20% experience mild flu-like symptoms referred to as West Nile fever [7]. A small percentage of the cases, less than 1%, can develop West Nile neuro-invasive disease, which is a more severe condition characterized WNV meningitis, encephalitis, or poliomyelitis [8, 9]. WNV has caused human and animal infections, and some fatal cases, particularly in America [10] and Europe [11, 12]. WNV has been classified into nine lineages (lineage 1 to lineage 9) based on biology, evolution, pathogenicity, and geographic distribution [13]. WNV lineages 1, 2, and the West Nile virus Koutango lineage (WN-KOUTV)—lineage 7 exhibit the highest virulence among the West Nile virus lineages. However, only lineages 1 and 2 have caused numerous outbreaks, sometimes accompanied by severe neurological disease [11]. In Kenya, West Nile Lineage 1a has been documented through isolation from field-collected mosquitoes [14, 15] and *Rhipicephalus pulchellus* ticks [16], as well as through serological evidence [17–19] though epidemics have not been reported. Recently WN-KOUTV was isolated from phlebotomine sandflies in Baringo county [20].

WN-KOUTV, predominately found in Africa, was for many years reported as a distinct flavivirus. However, based on phylogenetic evidence, it is presently regarded as a distant variant of the WNV [21, 22]. WN-KOUTV takes its name from the Koutango district of the Kaolack region in Senegal, where the virus was first recovered from the wild rodent *Tatera kempi* in 1968 [23]. Subsequently, it was detected in M*astomys sp*. 1974 in Senegal and later detected in *Lemnyscomys sp*. in Central African Republic [24]. In addition, it has been isolated from a range of arthropods including mosquitoes, ticks, and phlebotomine sandflies [24]. In animal models, investigations done on mice have consistently showed that WN-KOUTV is the most neurovirulent WNV lineage [25–27]. Potential risk for humans has been highlighted following an accidental infection of a Senegalese lab worker characterized by fever and rash [28]. There is also documented serological evidence in humans in Gabon [29] and Sierra Leone [30]. *Culex spp*. primary competent vectors of WNV lineage 1 and 2 were found to be incompetent vectors of WN-KOUTV [31]. However, trans-ovarially transmission has been documented in *Aedes Aegypti* [32]. Also, dissemination of WN-KOUTV by *Aedes aegypti* was demonstrated, but only at high titers [33].

Despite these data WN-KOUTV remains a neglected arbovirus with lots of knowledge gaps regarding its persistence, transmission and maintenance cycle, genetic diversity, competent vectors, and hosts. Also, the role of phlebotomine sandflies in its maintenance or transmission is unknown.

Phlebotomine sandflies (Diptera: Psychodidae, Phlebotominae) are of medical-veterinary relevance in the tropics and subtropics, mainly due to their role as potent vectors of Leishmania parasites, the causative agents of leishmaniases [34]. These insects are also involved in the transmission of several arboviruses (genera Phlebovirus, Vesiculovirus, and Orbivirus), which are associated with febrile illnesses [35, 36], and are the vectors of the bacterium Bartonella bacilliformis, which causes Carrion’s disease [37]. However, there is paucity of data associating transmission WN-KOUTV to phlebotomine sandflies. Therefore, this study aimed to characterize WN-KOUTV in female phlebotomine sandflies, from study sites in Baringo and Isiolo Counties of Kenya. The information gained from this study sheds light on the circulation of WN-KOUTV as well as its genetic epidemiology and the nature of *in vitro* growth kinetics when compared to WNV lineage 1a.

## Results

### Phlebotomine sandflies collection and virus isolation

**Fig 1:**
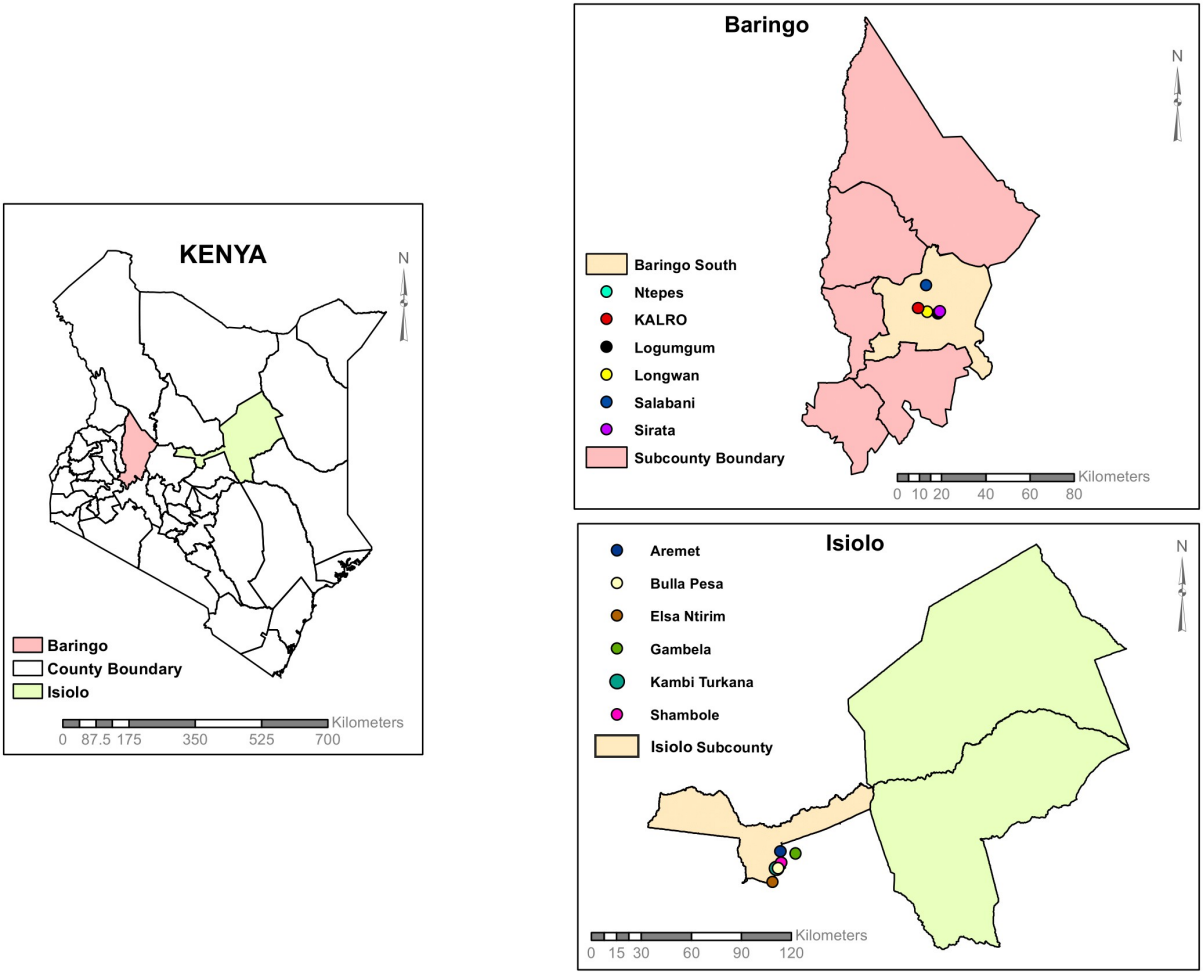
Map of Kenya, Isiolo and Baringo Counties showing the sampling sites in this study. The map was developed using ArcGIS Software Version 10.2.2 (http://desktop.arcgis.com/en/arcmap) advanced license.

A total of N = 7,737 female phlebotomine sandflies were collected in March 12^th^-16^th^ 2021 and 16^th^ -20^th^ March 2023, from study sites in Baringo South and Isiolo Sub-County, Kenya (Table 1 and Fig 1). They were organized into 536 pools according to genus and site of collection. Pools BAR_S3_S008 and ISL_S6_2050, which consisted of *Sergentomyia spp*. sandflies trapped in Salabani village (Lat/Lon N0.56187 E36.03038, altitude 3289 ft) and Aremet village (Lat/Lon N0.44071 E37.58164, altitude 3281 ft), respectively, exhibited clear cytopathic effect (CPE). The CPE was characterized by rounding of cells, alterations in cell morphology, and disruption of the monolayer, starting on day 3 post-inoculation and consistently observed in subsequent passages.

**Table 1:** Distribution of phlebotomine sandflies collected from study sites in Baringo South and Isiolo using CDC miniature light traps.

Locality	No. of traps/12 h	Number of *Sergentomyia spp*	Number of *Phlebotomus spp*	Total
Salabani	12	*389*	*10*	399
Longumgum	12	109	21	130
Sirata	12	35	5	40
Ntepes	12	479	58	537
Longwan	12	437	24	461
KALRO	12	2,078	34	2,112
Kambi Turkana	12	240	4	244
Aremet	12	1,377	6	1,483
Gambela	12	1,148	2	1,150
Elsa Ntirim	12	561	8	569
Shambole	12	285	0	285
Bulla pesa	12	427	0	427
**Total Phlebotomine sandflies**				**7,837**

### Genetic characterization of the WN-KOUTV isolates

The complete genomes of the BAR_S3_S008 and ISL_S6_2050 isolates were determined. The genomes comprise 10,924 and 10,998 nucleotides, respectively, with a G+C content of 50.6%. Sequencing achieved an average depth of coverage of 3864x and 10058x, respectively (S1 Fig). These genomes contain a single open reading frame spanning 10,314 nucleotides: from nucleotide positions 30 to 10,343 for BAR_S3_S008 and from 107 to 10,420 for the ISL_S6_2050 isolate, encoding a polyprotein of 3437 amino acids. BLASTn analysis identified the genomes as belonging to the flavivirus West Nile virus Koutango lineage. Nucleotide similarity to available ORF sequences of other WN-KOUTV strains ranged between 89.85% to 99.25%, and were 98.20% similar to each other, while amino acid identity ranged from 97.42% to 99.71% with other WN-KOUTV strains and 99.53% similar to each other (Table 2).

**Table 2:** Nucleotide and amino acid identity rates between WN-KOUTV BAR_S3_S008 and ISL_S6_2050 and other WN_KOUTVs.

Strain	Genbank Accession Number	Origin	Genome (nt)	Date of Isolation	Host	ISL_S6_2050		BAR_S3 _S008	
						nt %	aa %	nt %	aa %
PM148	MN057643.1	Niger	10,948	2016	Phlebotomine Sandflies	89.85	98.34	90.11	98.52
ArD96655	KY703855.1	Senegal	10,302	1993	*Rhipicephalus guilhoni*	89.92	98.05	90.15	98.23
AnD5443	EU082200.2	Senegal	10988	1968	*Tatera Kempi*	90.95	98.40	91.20	98.49
AnD95153	OP846972.1	Senegal	10,104	1993	*Mastomys erythroleucus*	89.86	97.42	89.98	97.63
BAR_SS4_020	ON158112.1	Kenya	9,826	2015	Phlebotomine Sandflies	98.78	99.33	99.25	99.71

### Phylogeny of the WN-KOUTV isolates

**Fig 2:**
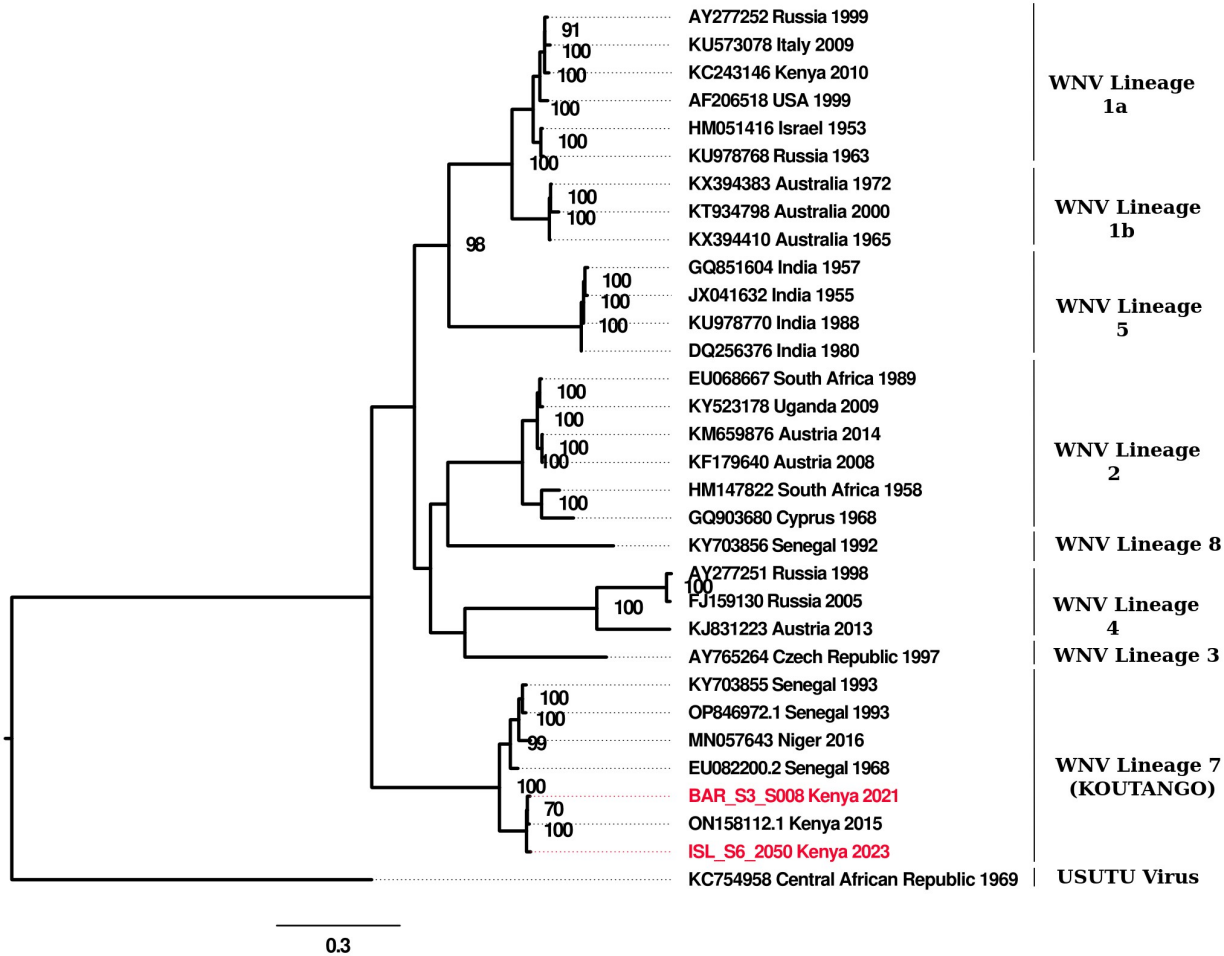
Maximum-likelihood phylogenetic tree showing the relationship of the WN-KOUTV strains BAR_S3_S008 and ISL_S6_2050, with select WNV strains. The robustness of the tree was assessed using bootstrap analysis. Only bootstrap values >70% are displayed at important nodes. Usutu virus was considered as the out-group. The scale bar represents nucleotide substitutions per site.

Maximum likelihood phylogenetic analysis of WN-KOUTV strains BAR_S3_S008 and ISL_S6_2050, alongside representative strains from diverse WNV lineages, revealed their placement within a well supported monophyletic clade. This clade includes WN-KOUTV strain BAR_SS4_020 (GenBank accession number ON158112.1), isolated from Baringo County, Kenya, in 2015. BAR_S3_S008 shows a closer phylogenetic relationship to BAR_SS4_020 compared to ISL_S6_2050. Notably, this clade forms a sister lineage to West African WN-KOUTV strains, as illustrated in Fig 2.

**Fig 3:**
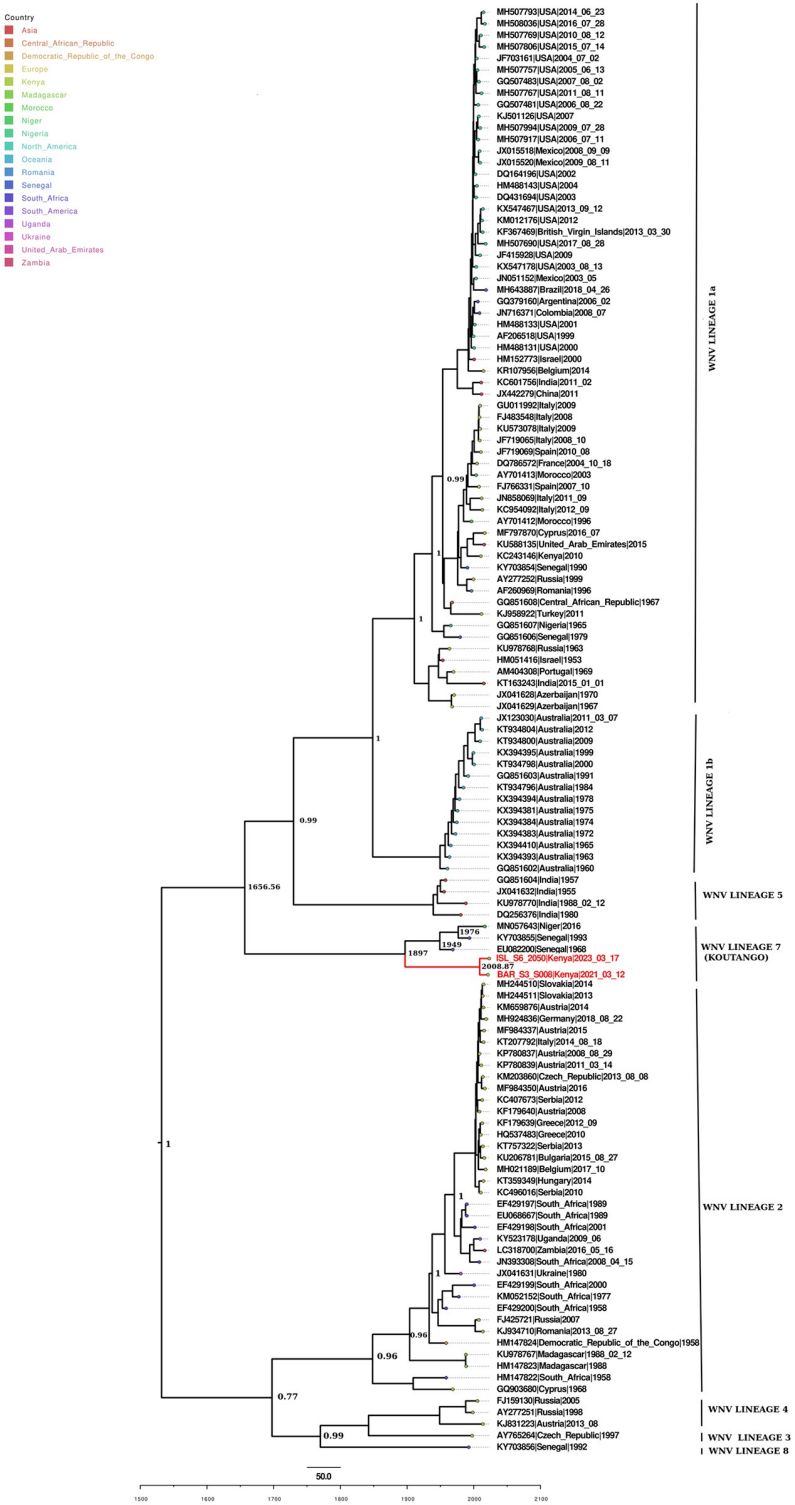
Time-calibrated phylogeny based on 125 genome sequences representing different WNV lineages from different countries. Tree nodes with a posterior probability greater than 0.7 are displayed. The current WN-KOUTV isolates strains are shown in red and tree tip-nodes are colored based on the inferred geographical location of origin for visual clarity. Branches are scaled in years before 2023.30.

The molecular clock analysis estimated the time to the most recent common ancestor (tMRCA) of West Africa and Kenyan WN-KOUTV to late 19th century (1897, 95% HPD: 1812.41, 1952.54), (Fig 3) making this the most likely latest time point of introduction of WN-KOUTV in the region. The tMRCA of BAR_S3_S008 and ISL_S6_2050 is more recently in 2008.87 (95% HPD: 1989.66, 2021.24). The estimated tMRCA of WN-KOUTV lineage was dated back to 1656.56.

### Amino acid variations among WN-KOUTV strains

**Fig 4:**
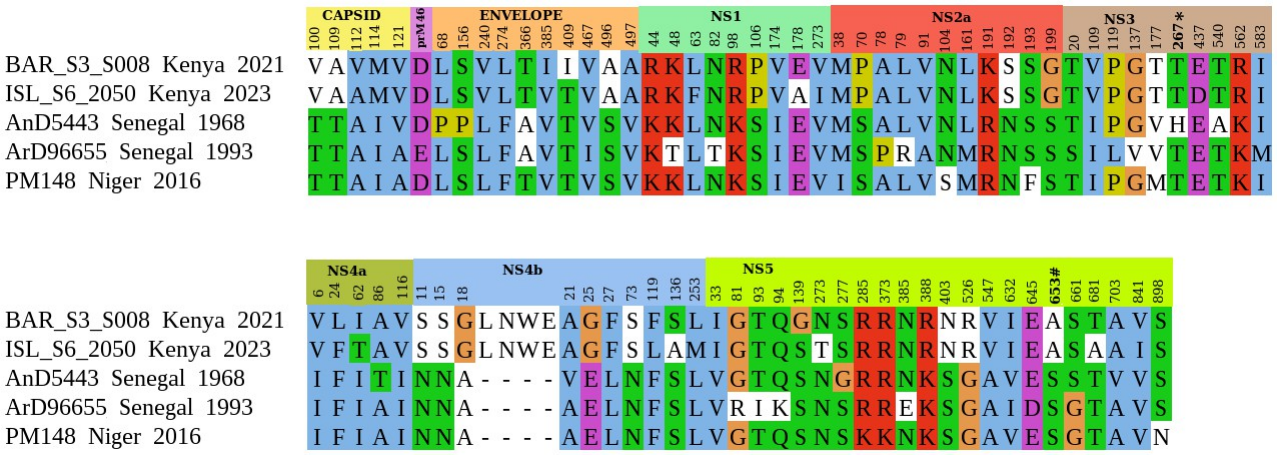
Amino acids variation among the WN-KOUTV strains. Amino acid variations mapped across WN-KOUTV strains polyproteins, revealing gene-specific differences.

The amino acid analysis of WN-KOUTV lineage strains’ open reading frame showed 87 out of 3437 (2.53%) variable sites among the strains. The NS2b gene is highly conserved without any variation in the amino acids (Fig 4). The Kenya WN-KOUTV strains showed 51 out of 87 (65.51%) amino acid variations in genes when compared to the other WN-KOUTVs. Amino acid variations in the Kenyan strains were found in the capsid gene 5(9.75%), envelope gene 6 (11.76%), NS1 gene 6(11.76%), NS2a gene 4 (7.84%), NS3 gene 3 (5.88%), NS4a gene 4(7.84%), NS4b gene 14 (10 amino acid variation and 4 amino acid insertion accounting for 27.45%), and NS5 gene 10 (19.60%) (Fig 4). The insertion of the 4 amino acids at NS4b resulted in a 3437 amino acid-long polyprotein, compared to the 3433-amino acid polyprotein observed in other WN-KOUTV strains. This characteristic is shared with a previous isolate from Kenya, strain BAR_SS4_020.

**Fig 5:**
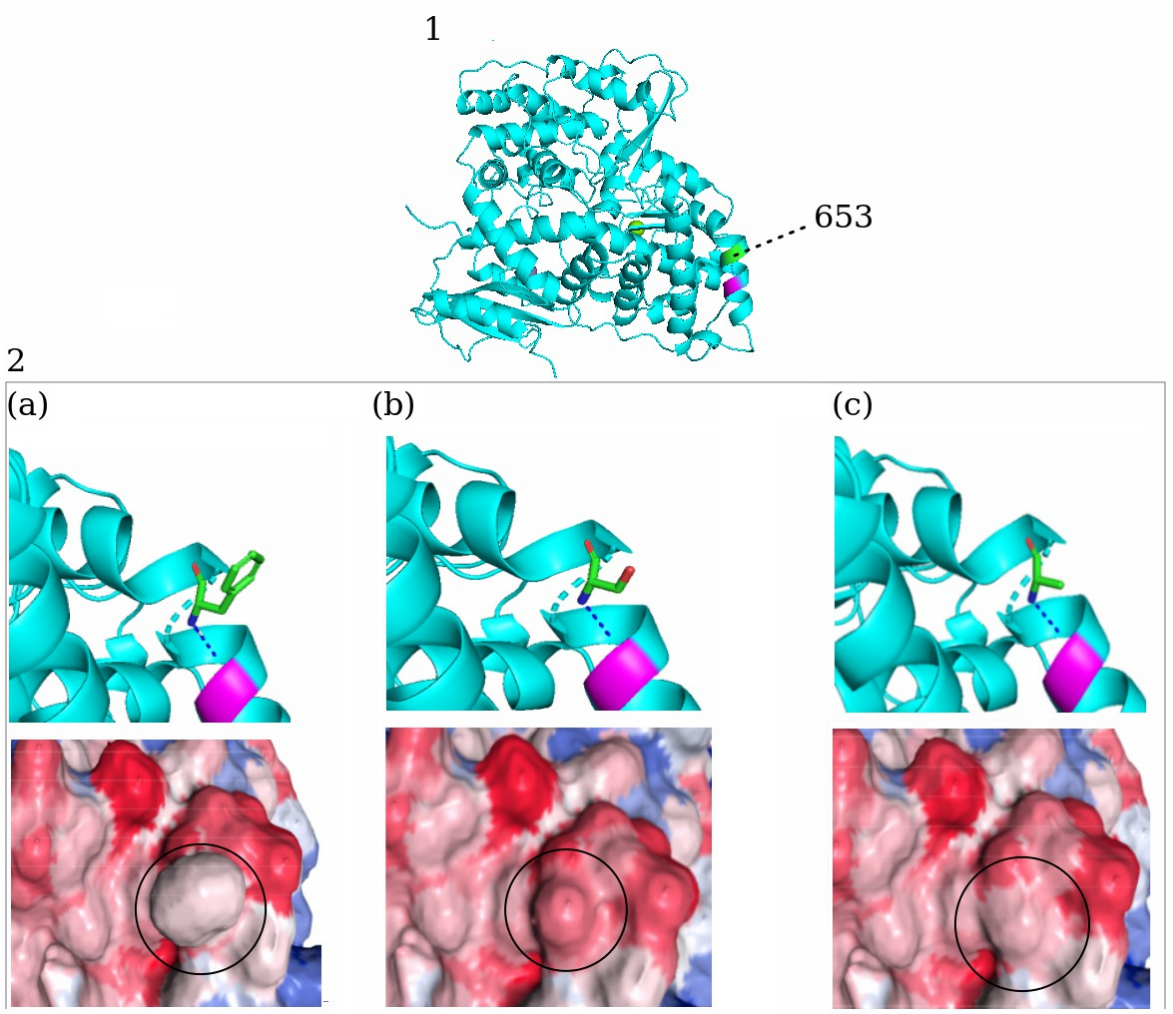
Amino acid variation at site 653 within NS5 protein. 1. West Nile virus NS5 protein showing residue 653 colored in green and closely associated amino acid (Magenta) mapped on the WNV KUN RdRp structure (Protein Data Bank ID: 2HFZ) [42]. 2. Close-up views of NS5 653: (a) Phenylalanine amino acid found in most WNV lineages strain, (b) Serine found in WN-KOUTVs from West Africa, and (c) Alanine found in WN-KOUTVs from Kenya, with their corresponding surface structure showing the surface charge distributions. Positive and negative electrostatic potential are indicated in blue and red, respectively, while white color represents neutral. Protein structure was modeled in PyMOL Version 2.6.0 (Schrödinger, LLC). Electrostatic potential was calculated using the pdb2pqr server and Adaptive Poisson-Boltzmann Solver (APBS) (v1.5). Images were generated using the NGL viewer.

Mutations characteristic of the WN-KOUTV lineage in reference to WNV NY99 strain, in published molecular determinants of WNV virulence and replication, were also observed in Kenya WN-KOUTV strains. These mutations include S72M in the pre-membrane, Y155F within the glycosylation site in the envelope, and a threonine (T) residue at position 249 of the NS3 protein [38–41]. However, alanine amino acid was observed at position 653 in the NS5 protein of the Kenyan WN-KOUTV strains while West Africa WN-KOUTV strains have Serine (Fig 5).

### Positive selection pressure was acting at codon site 1772-NS3:267 of WN-KOUTV lineage

A dataset consisting of five WN-KOUTV genomes was assessed for selection pressure. The overall ratio of non-synonymous to synonymous substitutions (dN/dS or ω) was determined to be 0.0235. Evidence of diversifying positive selection was observed at one site (1772-NS3:267), supported by three of the employed methods namely, MEME at p≤ 0.01, and FEL at p≤ 0.067 and BUSTED p≤ 0.003 (Table 2).

### WN-KOUTV replicated in Vero E6 and C6/36 cells at rates comparable to WNV lineage 1a

**Fig 6:**
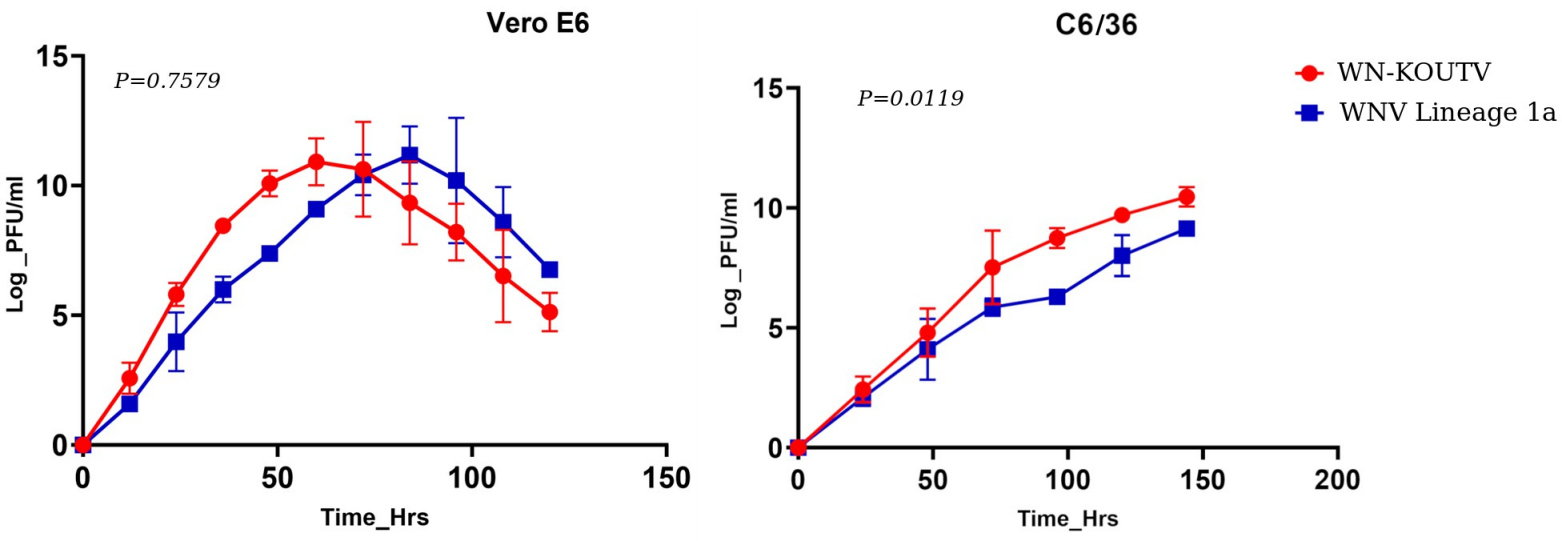
Comparative growth kinetics WN-KOUTV Strain BAR_S3_S008 and WNV Lineage 1a strain AMH005348 in (A) Vero cells and (B) C6/36 Mosquito cells. Viral titers were assessed on Vero E6 cells through plaque assay at specified time intervals and are expressed as Log PFU/mL. Graphs were generated using GraphPad Prism v10.1.2, and statistical significance was determined utilizing paired t-tests. The error bars represent the range of mean values obtained from duplicates in two independent experiments. *P < 0.05.

WN-KOUTV BAR_S3_S008 isolate showed good growth in both Vero and C6/36 cells, as indicated by the amount of infectious viral particles (PFU/ml) in the supernatant fraction. In Vero cells, the number of infectious viral particles of WN-KOUTV increased gradually to a maximum peak titer of 1×10^11 PFU/mL at 60 hours post-infection (hpi), before the titers began to decrease. In C6/36 mosquito cells, WN-KOUTV reached high titers (1×10^10 PFU/mL) within 144 hours of the assay. Overall, there was no significant difference in infectious viral particle production between the two WNV lineages in Vero cells despite WN-KOUTV reaching peak titers earlier than WNV lineage 1a. However, there was a significantly higher rate for WN-KOUTV in C6/36 cells (P < 0.0119) (Fig 6).

## Discussion

The presence of WN-KOUTV lineage in Africa have been documented through serological evidence [29, 30], isolation from mammals [23] and diverse arthropods [24]. Notably, its heightened neurovirulence in animal models compared to other WNV lineages [25–27] underscores the need for a deeper understanding of its circulation, genomic traits, and potential vectors. The recent isolations of WN-KOUTV from phlebotomine sandflies in Niger [24] and Kenya [20] highlights their possible role as vectors. The present study successfully isolated and obtained the complete genomes of the WN-KOUTV from phlebotomine sandflies sampled from Baringo and Isiolo Counties of Kenya. This subsequent detection of WN-KOUTV in geographically unlinked regions of Kenya suggests it widespread, continued circulation and highlights the potential involvement of phlebotomine sandflies, in maintaining the virus within its enzootic transmission cycle. Phlebtomine sandflies in the WN-KOUTV positive pool were morphologically identified as *Sergentomyia spp*. *Sergentomyia spp*. are widely distributed in Kenya [43] including in this regions as observed in Table 1. Traditionally, *Sergentomyia spp* are known for their preference to feed on cold-blooded animals [44, 45], however, recent studies have revealed DNA from various vertebrates, including humans, in blood-fed females of different Sergentomyia species, suggesting a potential vector role [46–48]. This isolations also emphasizes the possible wider range of vectors of WN-KOUTV, with previous isolations from mosquitoes and ticks in Senegal [25] and from phlebotomine sandflies in Niger in 2015 [24].

Our molecular clock inference revealed the most recent common ancestor of Kenyan and West African WN-KOUTVs dates to 1897 (95% HPD: 1812.41, 1952.54) (Fig 3) indicative of the period of possible introduction of WN-KOUTV in the region. The extended branch leading to the Kenya WN-KOUTV isolates underscores the historical continuity of the virus’s circulation and highlights potential gaps in surveillance data. This may be attributed to the limited attention given to phlebotomine sandfly-borne viruses in the Sub-Saharan region until recently [48, 49]. Historically, surveillance efforts have predominantly focused on vectors such as mosquitoes and ticks [50], potentially allowing the circulation of WN-KOUTV to go undetected for an extended period. Notably, WNV lineage 1a, primarily transmitted by mosquitoes, has been documented through serological evidence in this region [19]. The two Kenyan strains are estimated to have diverged more recently in 2008.87 (95% HPD: 1989.66–2021.24), indicating ongoing evolutionary dynamics possibly influenced by local environmental factors or vector dynamics. The estimated tMRCA of the WN-KOUTV lineage was traced back to 1656.56, underscoring its long-standing presence and evolutionary history. Given that birds serve as the natural reservoir hosts for WNVs [6], and considering the documented adaptation of WN-KOUTV to avian cells [51], along with the observed phylogenetic relationship of West and East Africa WN-KOUTV (Fig 3), we speculate that intra-African migratory birds between these regions may potentially have contributed to the movement of the virus between the two regions.

Similar to the West African WN-KOUTVs, Kenya WN-KOUTVs strains lack any known published virulence motifs found in the WNV NY99 strain [24, 25, 27]. However, at position NS5 653 which is associated with interferon resistance, Kenya WN-KOUTV strains have alanine amino acid compared to serine found in the West African WN-KOUTV strains (Fig 5). In the naturally attenuated WNV Kunjin subtype, site-specific mutagenesis studies at NS5 653, where serine is replaced by phenylalanine as in WNV NY99, increased sensitivity to type I interferon-mediated JAK-STAT signaling, revealing the role of site NS5 653 in interferon suppression, potentially enhancing the virus’s virulence [52]. Amino acid changes at this site represents a change in hydrophobicity and charge distribution as shown in Fig 5. Therefore, further studies are needed to the determine implications of A653 in Kenya WN-KOUTV strains on interferon resistance.

This study has found purifying pressure acting on WN-KOUTV (Table 3), aligning with observations in other vector-borne RNA viruses. This may be attributed to the interaction of the arbovirus with its dual hosts, the arthropod, and the vertebrate, each equipped with distinct defense mechanisms [53]. Nevertheless, we found evidence of diversifying pressure at codon site 1772 of the polyprotein (NS3 267). NS3 267 is located within the NS3 helicase domain, which is crucial to viral replication [54]. However, no documented role has been attributed to this site in WNV. Therefore, further investigation may elucidate the precise functional implication of this variation.

**Table 3:** Selection pressure analysis table indicating positively selected site 1772 (NS3:267) of WNV-KOUTV lineage.

West Nile Virus Dataset	Overall ω value	Codon	Protein and amino-acid position	Associated amino-acid substitution	Methods		
					MEME	FEL	BUSTED
					P-value		
**Lineage 7 (Koutango) n = 5**	0.0235	1772	**NS3:267**	T→H	0.01	0.067	0.003

Our study demonstrated growth of WN-KOUTV in both mammalian Vero E6 cells and mosquito cell lines C6/36, comparable to WNV lineage 1a (Fig 6) indicating its adaptability to mammalian and mosquitoes cell lines despite being isolated from sandflies. Whereas there was no significant difference in infectious particles production between WN-KOUTV and WNV lineage 1a in Vero E6 cells, a significantly higher rate for the WN-KOUTV in C6/36 cells was observed (p-value = 0.0119), in agreement with Fall et al. (2017) [25]. Notably, although the WN-KOUTV replicated well in mosquito cells, the virus has not yet been isolated from mosquitoes in Kenya. Interestingly, dissemination of DAK Ar D 5443 WN-KOUTV strain by *Aedes aegypti* was demonstrated at only high virus titers [33]. Therefore, the re-isolation of WN-KOUTV from disparate sites in Kenya in phlebotomine sandflies emphasizes the need for further studies to determine the role of these vectors in the transmission dynamics of the virus. Additionally, there is a need to gather serological evidence across avian, human, and other mammalian populations to better understand the epidemiology and potential health impacts of WN-KOUTV.

## Materials and methods

### Ethics statement

The study received approval from the Kenya Medical Research Institute (KEMRI) Scientific and Ethics Review Unit under protocols number 3948 and 4570. It was exempted from review by the Walter Reed Army Institute of Research (WRAIR) Human Subjects Protection Branch, as per WRAIR Policy 25, as there is no human subjects data or samples being analyzed. Additionally, verbal consent was obtained from the heads of households to sample on their farms.

### Study area

The study area encompassed a total of twelve villages: six from Baringo South Sub-County, Baringo County, and six from Isiolo Sub-County, Isiolo County, Kenya (Fig 1). Baringo South is situated in the Rift Valley region of Kenya and is one of the sub-counties within Baringo County. Baringo South covers an area of 1,985.11 km^2^ and has a population of approximately 90,388 people according to the 2019 census whose primary economic activity is agro-pastoralism.

Isiolo Sub-County is situated in Isiolo County, strategically positioned between Northern and Southern Kenya. It is one of the three sub-counties within Isiolo County and covers an area of 2,691 km^2^. According to the Kenya National Bureau of Statistics report of 2019, Isiolo Sub-County has a total population of 121,061 people. Isiolo Sub-County lies within two ecological zones: semi-arid and arid. The primary economic activity in Isiolo Sub-County is pastoralism, along with trade in Isiolo town. The choice of study areas was informed by the history of febrile illness outbreaks, previous cases or reports of human fevers of unknown origin, the presence of previously reported arboviruses including WN-KOUTV, and the abundance of phlebotomine sandflies. The map of the study area was developed using ArcGIS Software Version 10.2.2 (http://desktop.arcgis.com/en/arcmap) advanced license.

### Phlebotomine sandflies sampling

Adult phlebotomine sandflies were sampled using CDC miniature light traps (Model 512, John Hock Co., Gainesville, Florida, USA). Traps were deployed overnight (6pm-6am) in favourable habitat, including animal sheds and termite mounds. Captured sandflies were temporarily immobilized using triethylamine, sorted, preserved in liquid nitrogen, and transported to the laboratory at the Kenya Medical Research Institute in Kisumu, where they were stored at -80°C until further processing. All sandflies collected were sorted based on their sex, and specifically, female sandflies were morphologically identified to the genus level using established keys. (Abonnenco, 1951; Kirk & Lewis, 1951). Subsequently, they were pooled in 1.5mL tubes, with a maximum of 10 specimens per pool, based on genus, location, trapping site, and capture day, and stored at -80°C.

### Sandflies homogenization

The sandfly pools were homogenized using a Mini-Beadruptor-16 (Biospec, Bartlesville, OK, USA) with zirconium beads (2.0 mm diameter) for 40 seconds. The homogenization was done in 500 μL of homogenization media, which consisted of minimum essential media supplemented with 15% fetal bovine serum (FBS) (Gibco by Life Technologies, Grand Island, NY, USA), 2% L-glutamine (Sigma, Aldrich), and 2% antibiotic/antimycotic (Gibco by Life Technologies, Grand Island, NY, USA). Subsequently, the homogenate was centrifuged at 10,000 rpm for 10 minutes at 4°C using a benchtop centrifuge (Eppendorf, USA), and the supernatant was collected.

### Virus isolation

Virus isolation was performed in Vero (African green monkey kidney) cells (CCL-81^™^), passage 13, obtained from the Viral Haemorrhagic Fever lab at KEMRI, as described before [55]. Briefly, Vero cells grown overnight at 37°C and 5% CO_2_, in minimum essential medium supplemented with 2% glutamine, 2% penicillin/streptomycin/amphotericin, 10% fetal bovine serum, and 7.5% NaHCO_3_ in 24-well plates (Corning, Incorporated). At 80% confluence, a 50 μL aliquot of the clarified supernatant from individual pools was inoculated into the wells. The plates were incubated for 1 hour in a humidified incubator at 37°C and 5% CO_2_, with gentle rocking of the plates side to side every 15 minutes for virus adsorption. Following incubation, 1mL of maintenance medium, comprising minimum essential medium supplemented with 2% glutamine, 2% penicillin/streptomycin/amphotericin, 2% fetal bovine serum, and 7.5% NaHCO_3,_ was added. The plates were then cultured in a humidified incubator at 37°C and 5% CO_2_, and monitored daily for CPE for 14 days [16]. Cultures exhibiting CPE were harvested and further passaged by inoculating them onto fresh monolayers of Vero cells (CCL-81^™^) in 25-cm^2^ cell culture flasks. After two successive passages, the supernatants of virus-infected Vero cell cultures exhibiting CPE of approximately 70% were harvested from the flasks for virus identification through next generation sequencing.

### Library preparation and next-generation sequencing

Viral particles from CPE positive cultures were recovered through 0.22μm filters (Merck Millipore, Darmstadt, Germany). From an aliquot of 140 μl of the supernatant, viral RNA was extracted using the QIAamp Viral RNA Mini Kit (Qiagen, Hilden, Germany) and eluted in one step with 60 μl of elution buffer. The extracted nucleic acid was quantified using the Qubit RNA fluorometer (Thermo Fisher Scientific, Waltham, Massachusetts, USA). Sequencing libraries were prepared using Illumina RNA Prep with Enrichment, Tagmentation Kit (Illumina, USA) following the manufacturer’s instructions. The obtained libraries were assessed for quantity using the Qubit 2.0 Fluorometer (ThermoFisher, Waltham, MA, USA). Sequencing was performed on an Illumina iseq 100 platform (Illumina, San Diego, California, USA) using iSeq 100 i1 Reagent v2 (Illumina, San Diego, California, USA) in a 2 × 150 bp paired-ended configuration.

### Sequence analysis

Raw sequencing data was initially processed for quality using Trimmomatic v0.39 [56]. This step involved the removal of adapters, duplicates and low-quality sequences. Sequences with phred scores ≥ 30, were used for further analysis. The cleaned reads were then assembled using Megahit v1.2.9 [57] with various k-mer values to reconstruct the viral genome. To identify viral contigs within the assembly, a BLASTn [58] search was conducted against the NCBI databases (https://blast.ncbi.nlm.nih.gov/Blast.cgi). The identified genomes was deposited in GenBank under the accession number PP489328 and PP885725. To determine the sequencing depth of the obtained genomes, sequencing reads were mapped against the genome using Bowtie2 v2.5.2 [59], and the depth of coverage were determined using Samtools depth v1.13.

### Phylogenetic analysis

Complete genomes representing all WNV lineages were retrieved from the Bacterial and Viral Bioinformatics Resource Center (BV-BRC) https://www.bv-brc.org/ and combined with the genome sequences of the isolated viruses. Alignment was performed using MAFFT v7.490 [60] and manually curated using BIOEDIT [61]. Phylogenetic analysis was conducted using the Maximum Likelihood method in IQTREE v2.0.7 [62] using GTR+F+I+G4 (General Time Reversible + Frequencies + Invariant Sites + Gamma distribution with 4 rate categories) substitution model as determined using Model-finder [63]. The robustness of the tree topology was assessed using 1000 non-parametric bootstrap analyses [64]. The resulting tree was annotated and visualized in FigTree v.1.4.4 (available from http://tree.bio.ed.ac.uk/software/figtree/).

### Molecular clock analysis

To estimate coalescent times of most recent common ancestor (tMRCA) for the WN-KOUTV isolates, molecular clock analysis was performed using the Bayesian Markov Chain Monte Carlo (MCMC) method implemented in BEAST v2.1.3 software package [65]. Representative complete genomes with country and year of isolation data, for all WNV lineages spanning continents worldwide were obtained from the Bacterial and Viral Bioinformatics Resource Center (BV-BRC) https://www.bv-brc.org/. These genomes were added to the dataset for maximum likelihood phylogenetic analysis, resulting in a total of (n = 125) genomes with an alignment length of 10,302 base pairs. The analysis employed the uncorrelated lognormal relaxed molecular clock model to account for rate variation across branches in the phylogenetic tree. Geographical traits were assigned to the sequences, and a flexible non-parametric Bayesian Skyline model and an asymmetric trait model were used to estimate past population dynamics and ancestral states, respectively. The MCMC analyses were run for 800 million states and sampled every 80,000th generations. Tracer v1.7.1 [66] was employed to assess the logs and ensure adequate effective sample sizes (ESS) values > 200 by visual inspection of chains. Maximum clade credibility (MCC) tree was generated using TreeAnnotator v2.1.2, discarding the initial 10% as burnin. The resultant phylogeny was visualized using FigTree v.1.4.4 (available from http://tree.bio.ed.ac.uk/software/figtree/) showing posterior probability values in each node and the time to the most recent common ancestors (tMRCA) as median year with 95% Highest Posterior Density (HPD).

### Amino acid variations analyses and computational protein modeling

Comparative amino acid analyses were performed using a dataset containing 5 WN-KOUTV complete coding regions. Amino acid alignment of the was performed using MEGA11 [67], showing only variable sites in each WN-KOUTV gene. The amino acid variation at the site of interest was mapped onto the Crystal structure of RNA dependent RNA polymerase domain from West Nile virus (Protein Data Bank ID: 2HFZ) [42], using the PyMOL Molecular Graphics System, Version 2.6 Schrödinger, LLC. Electrostatic surface potential was calculated using the pdb2pqr server and APBS (v1.5) [68]. Graphical representations of electrostatics surface potential were generated using NGL viewer [69].

### WN-KOUTV lineage selection pressure analyses

Evolutionary pressures acting across the entire coding sequence of WN-KOUTV lineage were assessed using various methods, including fixed-effects likelihood (FEL), single-likelihood ancestor counting (SLAC), mixed effects model of evolution (MEME), Branch-site Unrestricted Statistical Test for Episodic Diversification (BUSTED) and fast unconstrained Bayesian approximation (FUBAR) on the Datamonkey selective and evolutionary bioinformatics online server [70]. To determine the selective pressure, the ratio (ω) of the rate of non-synonymous substitutions (dN) to the rate of synonymous substitutions (dS) per codon site was calculated using the SLAC and FEL codon-based maximum likelihood approaches. A value of ω ≥ 1 indicates positive selection, ω ≤ 1 indicates negative selection, and ω = 0 indicates neutral selection. Significance level was set at p≤0.1 for FEL, SLAC, and MEME, posterior probability at ≥ 0.9 for FUBAR and p≤ 0.05 for BUSTED as recommended [71]. Positive selection at a specific site was considered present when at least three methods detected it.

### Time course infection of Vero E6 and C6/36 cells with WN-KOUTV isolate in comparison to West Nile virus lineage 1a

To compare WN-KOUTV isolate with West Nile Lineage 1a growth kinetics in mammalian and mosquito cell lines. Time course infection of Vero E6 (Chlorocebus sabaeus) cells and C6/36 cells (*Aedes albopictus*) was carried out as previously described in duplicates [25]. Briefly, virus stocks of WN-KOUTV BAR_S3_S008 and the previously isolated Kenyan WNV Lineage 1a strain (AMH005348) were generated from passage 3 in Vero E6 cells (Sigma Aldrich, France), and used in this assay. The viral stocks titers were determined using the Vero plaque assay, resulting in titers of 1.1x10^^6^ and 1x10^^6^ plaque-forming units (PFU) for WN-KOUTV and WNV lineage 1a, respectively. 2x10^^5^ Vero E6 cells and C6/36 cells were seeded in 24-well plates and incubated in a humidified incubator at 37°C and 5% CO_2_. At 80% confluency, cells were infected with 2x10^^3^ PFU of virus in 50 μl of maintenance medium, for a multiplicity of infection (MOI) of 0.01. After a 1-hour incubation period for virus adsorption, minimum essential medium supplemented with 2% glutamine, 2% penicillin/streptomycin/amphotericin, 2% fetal bovine serum, and 7.5% NaHCO_3_ was added, and this time point was set as the starting point for the growth curves (T0). The cells were then incubated in a humidified incubator at 37°C and 5% CO_2_ for Vero cells and at 28°C for C6/36 cells. The infectious supernatant was harvested every 12 hours for 5 days for Vero cells and every 24 hours for C6/36 cells for 6 days. All collected samples were stored at -80°C, and subsequent viral titers were determined using the plaque assay method. Two independent experiments were performed to determine the reproducibility of the results.

### Infectious virus quantification

Infectious WN-KOUTV and WNV lineage 1a were quantified via plaque assay. Briefly, 2.3x10^^6^ Vero E6 cells were seeded in 12 well plates a day prior the infection. Supernatants were serially diluted 1:10 in maintenance media before inoculation of 100ul into 75–95% confluent cells. Plates were incubated at 37°C and 5% CO_2_ for 1 h for virus adsorption with rocking back and forth every 15 mins. Following the adsorption process, 2.5% methylcellulose overlay was added to each well and plates incubated at 37°C and 5% CO_2_ for six days. Methylcellulose was aspirated out and cells fixed with 3.7%v/v formaldehyde (Sigma) overnight and stained overnight with 0.5% crystal violet (Sigma) diluted in absolute ethanol. The plates were then soaked in tap water for 10 minutes and to remove excess stain and allowed to dry followed by manual counting of plaques.

### Statistical analysis

Mean viral titers from duplicates per independent experiment were calculated and expressed as Log PFU/ml. Paired t-test analyses were performed using GraphPad Prism v10.1.2 (GraphPad Diego, CA, USA, 2016) at all-time points to determine significant difference in infectious viral particles production between WN-KOUTV and WNV lineage 1a within the same cell line, with a significance threshold of p < 0.05.

## Conclusion

Our study expands the understanding of WN-KOUTV in Africa, highlighting its widespread circulation and potential involvement of phlebotomine sandflies as vectors. The isolation from phlebotomine sandflies suggests its adaptive capability and indicates a broader range of potential vectors beyond mosquitoes and ticks. Additionally, its replication in both mammalian and mosquito cell lines emphasizes its adaptability across various hosts and transmission dynamics. Molecular clock analyses revealed the longstanding presence and evolutionary dynamics of WN-KOUTV in the region, underscoring the need for enhanced surveillance efforts in other parts of Africa to assess the presence and potential spread of WN-KOUTV. Further characterization of the virus will enhance our understanding of the risks it poses and inform strategies for its prevention and control.

## Supporting information

S1 Fig Plot showing genome depth of coverage for the isolated WN-KOUTVs A) WN-KOUTV strain BAR_S3_S008 and B) WN-KOUTV strain ISL_S6_2050. (TIFF)

S1 File Growth kinetics raw data. (PDF)

S2 File Beast files used for generating time calibrated Bayesian phylogeny. (ZIP)
