# Supplementary figures and images for: Characterization of West Nile virus Koutango lineage from phlebotomine sandflies in Kenya
Source: PLoS One. 2024 Aug 22;19(8):e0301956. doi: 10.1371/journal.pone.0301956 (PMC11341046; doi:10.1371/journal.pone.0301956)

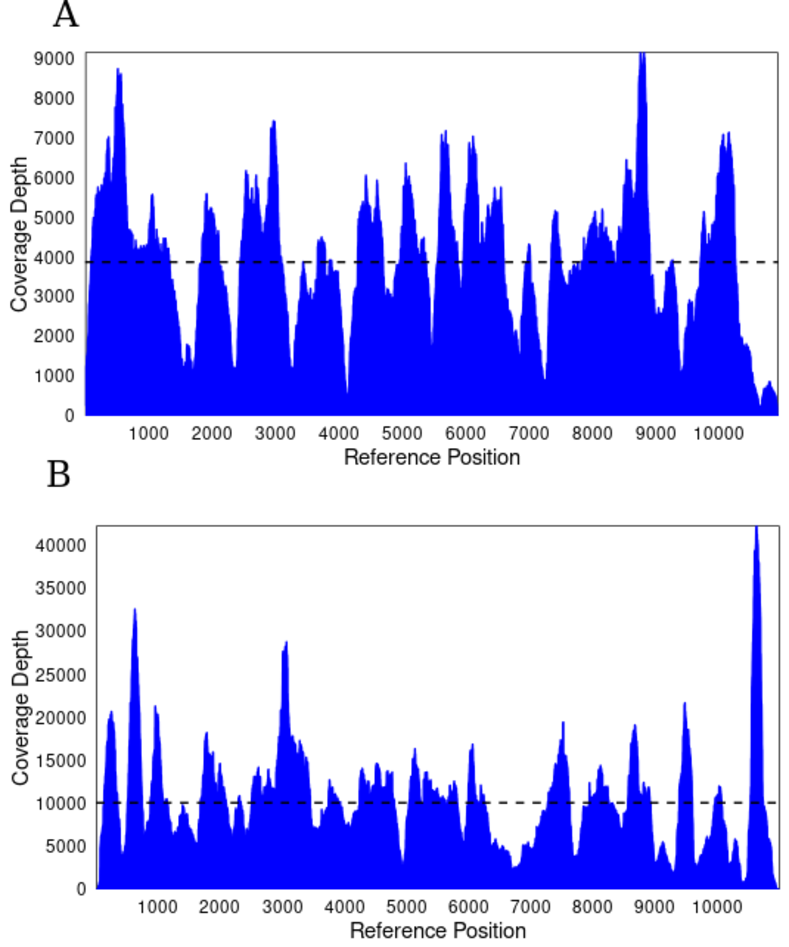

Supplement: S1 Fig — (TIFF) [file pone.0301956.s001.tiff]
