# Supplementary material for: Characterization of West Nile virus Koutango lineage from phlebotomine sandflies in Kenya
Source: PLoS One. 2024 Aug 22;19(8):e0301956. doi: 10.1371/journal.pone.0301956 (PMC11341046; doi:10.1371/journal.pone.0301956)
Supplement: S1 File — (PDF) [file pone.0301956.s002.pdf]

TABLE 1: Number of plaques forming unit (PFU)/ml at each time point (VERO E6 Cells)

Time	Number of plaques forming unit (PFU)/ml			
	West Nile virus-Koutango Lineage	West Nile virus-Koutango Lineage Replicate 1	West Nile virus Lineage 1a	West Nile virus Lineage 1a Replicate 1
**0 hrs**	0	0	0	0
**12hrs**	$1.4x10^{2}$	$1x10^{3}$	$5x10^{1}$	$3.x10^{1}$
**24hrs**	$3.1x10^{5}$	$1.3x10^{6}$	$1.5x10^{3}$	$6x10^{4}$
**36hrs**	$2.5x10^{8}$	$3.2x10^{8}$	$4.4x10^{5}$	$2.2x10^{6}$
**48hrs**	$2.7x10^{10}$	$5.4x10^{9}$	$1.8x10^{7}$	$3.3x10^{7}$
**60 hrs**	$1.9x10^{10}$	$3.6x10^{11}$	$1.2x10^{9}$	$1.3x10^{9}$
**72hrs**	$2.2x10^{8}$	$8.3x10^{11}$	$7.2x10^{9}$	$9.2x10^{10}$
**84hrs**	$1.6x10^{8}$	$2.9x10^{10}$	$2.5x10^{10}$	$6.0x10^{11}$
**96hrs**	$2.7x10^{7}$	$9.6x10^{8}$	$3.1x10^{8}$	$8.4x10^{11}$
**108 hrs**	$1.8x10^{5}$	$6x10^{7}$	$4.3x10^{7}$	$3.5x10^{9}$
**120hrs**	$4x10^{4}$	$4.5x10^{5}$	$6.9x10^{6}$	$4.8x10^{6}$

TABLE 2: Number of plaques forming unit (PFU)/ml at each time point (C636 Cells)

Time	Number of plaques forming unit (PFU)/ml			
	West Nile virus-Koutango Lineage	West Nile virus-Koutango Lineage Replicate 1	West Nile virus Lineage 1a	West Nile virus Lineage 1a Replicate 1
**0 hrs**	0	0	0	0
**24hrs**	$6.4x10^{2}$	$1.1x10^{2}$	$2.2x10^{2}$	$7x10^{1}$
**48hrs**	$3.2x10^{5}$	$1.2x10^{4}$	$1x10^{5}$	$1.6x10^{3}$
**72hrs**	$4x10^{8}$	$2.7x10^{6}$	$1x10^{6}$	$4x10^{5}$
**96hrs**	$1.1x10^{9}$	$2.2 x10^{8}$	$2.7x10^{6}$	$1.4x10^{6}$
**120hrs**	$3.2x10^{9}$	$7.7x10^{9}$	$2.5x10^{7}$	$4.1x10^{8}$
**144hrs**	$5.6x10^{10}$	$1.5x10^{10}$	$8x10^{8}$	$2.0x10^{9}$
